# Deletion of *Cdk5* in Macrophages Ameliorates Anti-Inflammatory Response during Endotoxemia through Induction of C-Maf and Il-10

**DOI:** 10.3390/ijms22179648

**Published:** 2021-09-06

**Authors:** Pauline Pfänder, Ann-Kathrin Eiers, Ute Burret, Sabine Vettorazzi

**Affiliations:** 1 Institute of Comparative Molecular Endocrinology (CME), Faculty of Natural Sciences, Ulm University, 89081 Ulm, Germany; p.pfaender@dkfz-heidelberg.de (P.P.); ann-kathrin.eiers@uni-ulm.de (A.-K.E.); ute.burret@uni-ulm.de (U.B.); 2 DKTK Brain Cancer Metabolism Group, German Cancer Research Center (DKFZ), Faculty of Bioscience, Heidelberg University, 69120 Heidelberg, Germany

**Keywords:** macrophage, Cdk5, c-Maf, Il-10, anti-inflammation

## Abstract

Immune response control is critical as excessive cytokine production can be detrimental and damage the host. Interleukin-10 (Il-10), an anti-inflammatory cytokine produced primarily by macrophages, is a key regulator that counteracts and controls excessive inflammatory response. Il-10 expression is regulated through the transcription factor c-Maf. Another regulator of Il-10 production is p35, an activator of the cyclin-dependent kinase 5 (Cdk5), which decreases Il-10 production in macrophages, thus increasing inflammation. However, Cdk5 regulation of c-Maf and the involvement of Il-10 production in macrophages has not yet been investigated. We used in vitro primary bone marrow-derived macrophages (BMDMs) lacking *Cdk5*, stimulated them with lipopolysaccharid (LPS) and observed increased levels of c-Maf and Il-10. In an in vivo mouse model of LPS-induced endotoxemia, mice lacking *Cdk5* in macrophages showed increased levels of c-Maf and elevated levels of Il-10 in lungs as well as in plasma, resulting in ameliorated survival. Taken together, we identified Cdk5 as a potential novel regulator of Il-10 production through c-Maf in macrophages under inflammatory conditions. Our results suggest that inhibition of Cdk5 enhances the c-Maf-Il-10 axis and thus potentiates improvement of anti-inflammatory therapy.

## 1. Introduction

Inflammation caused by invading pathogens activates the innate and adaptive immune response. Monocytes and macrophages play critical roles in the first line of defense. The activation of macrophages must be tightly controlled because excessive pro-inflammatory cytokine production can be detrimental towards the host. Macrophages counter-regulate the enhanced pro-inflammatory response by production of anti-inflammatory cytokines such as interleukin-10 (Il-10). Thus, the balance between the pro- and anti-inflammatory gene program, as well as the state of the macrophages, is crucial for the response of the host immune system.

Il-10, among other cytokines, is a potent anti-inflammatory cytokine with immunosuppressive functions that is produced by dendritic cells, T-cells, B-cells, and macrophages [1,2]. The crucial function of Il-10 as an immunoregulator in the intestinal tract was shown in Il-10-deficient mice that develop chronic enterocolitis and inflammatory reactions under non-specific pathogen-free conditions [3]. In addition, Il-10-deficient mice are extremely sensitive to low doses of LPS (5 µg/mouse) and have increased mortality during endotoxemia, a mouse model for sepsis, that is mitigated by Il-10 infusion [4]. A complication that can develop from sepsis is acute lung injury or acute respiratory distress syndrome (ARDS) [5]. In nearly 40% of sepsis patients, progression of an acute lung injury is observed, and half of these patients die from the resulting inflammatory lung disease [6,7]. Therefore, it is important to identify modulators and targets to resolve (lung) inflammation. Il-10 administration in endotoxemic mice prolonged survival and reduced extravascular lung fluid as well as inflammation in the lung compared to only LPS-treated animals [8], showing that Il-10 is crucial to ameliorate the immune response and inflammation in the lung.

In macrophages, Il-10 is regulated by c-Maf (V-maf musculoaponeurotic fibrosarcoma oncogene homolog) [9,10]. *c-Maf* knockout or siRNA mediated *c-Maf* knockdown in macrophages lead to an impaired Il-10 mRNA and protein production [9,10,11]. c-Maf belongs to the Ap1 family and is a basic leucine zipper transcription factor [12]. c-Maf binds to the c-Maf recognition elements (MARE motifs) in the Il-10 promoter, enhances the Il-10 production and thus exerts anti-inflammatory effects [13,14]. Furthermore, c-Maf inhibits Il-12 production that causes pro-inflammatory effects [9,10,15].

p35 (Cdk5r1) is another recently described regulator of Il-10 production in macrophages [16]. In primary macrophages, Toll-like receptor 4 (TLR4) stimulation increases p35 expression, which binds and activates the cyclin dependent kinase 5 (Cdk5) resulting in decreased Il-10 production [16]. Vice versa, *p35* knockout macrophages have enhanced Il-10 production after LPS stimulation [16]. Previous studies showed that total *p35* KO mice have an ameliorated colitis in the dextran sulfate sodium model and a reduced disease severity in an endotoxemia model through enhanced production of Il-10 [16]. Cdk5, which is activated by p35 and p39 [17], is a unique member in the Cdk family because it is a proline-directed serine and threonine kinase. In addition to the earliest description of the function of Cdk5 in the brain, the role of Cdk5 in immune cells, where it regulates neutrophil degranulation and T-cell activation, has been most notably reported in recent years [18,19]. In our previous study we could show in macrophages, that the pan-Cdk inhibitor roscovitine as well as macrophage specific *Cdk5* deletion enhance the anti-inflammatory effects of glucocorticoids on inducible nitric oxide synthase (iNos) and NO production after inflammatory stimuli [20]. This demonstrates a significant regulatory role of Cdk5 during inflammation.

Since it has already been shown that in inflammatory macrophages reduced p35 (Cdk5 activator) increases Il-10 production [16], and in another study that Il-10 production during inflammation is regulated by c-Maf [9,10] we wanted to investigate whether Cdk5 regulates the transcription factor c-Maf and thereby Il-10 production in macrophages. This is important to understand the mechanism(s) underlying the resolution of inflammation.

Here, we report a novel role for Cdk5 in the resolution of inflammation by regulating Il-10 production through c-Maf in primary macrophages. Inhibition of Cdk5 with roscovitine or in the absence of Cdk5, LPS-stimulated macrophages are more potent to induce c-Maf and its downstream effector Il-10 when compared to wild-type macrophages. Mice lacking *Cdk5* in macrophages (*Cdk5*^LysMcre^) show increased pulmonary c-Maf, Il-10, and plasma Il-10 levels in a model of LPS-induced endotoxemia, resulting in improved disease severity and survival.

These results suggest that during inflammation deletion of *Cdk5* induces c-Maf and thus Il-10 production in macrophages, contributing to an enhanced anti-inflammatory response which improves survival. Impairing Cdk5 activity, particularly in macrophages, may be a novel treatment strategy for inflammatory diseases.

## 2. Results

### 2.1. Inhibition of Cdk5 with Roscovitine or Cdk5 Genetic Deletion in Macrophages Enhances Il-10 Expression through C-Maf after Inflammatory Stimuli

**Figure 1:**
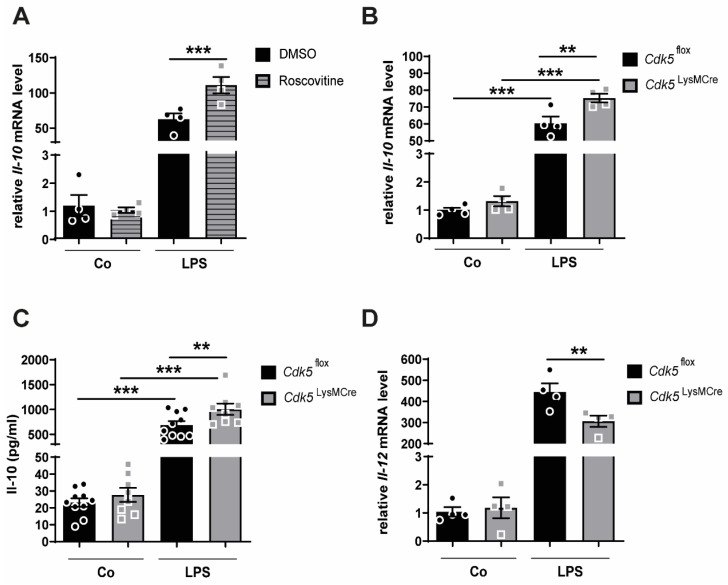
Inhibition or genetic deletion of Cdk5 in bone marrow-derived macrophages has an impact on the anti-inflammatory Il-10 and pro-inflammatory *Il-12* response. (**A**) BMDMs derived from wild-type mice were 30 min pre-treated with 0.16 µM roscovitine, the specific concentration for Cdk5 inhibition, or DMSO (vehicle control), and then stimulated 4 h with PBS (Co) or LPS (100 ng/mL) and relative expression of *Il-10* mRNA was determined by qRT-PCR. (**B**–**D**) BMDMs isolated from *Cdk5*^flox^ and *Cdk5*^LysMCre^ mice were stimulated with PBS (Co) or LPS (100 ng/mL) for 4 h and (**B**) relative *Il-10* mRNA expression, (**C**) Il-10 secretion into the supernatant and (**D**) *Il-12* mRNA expression were determined by qRT-PCR or ELISA. Results are depicted as mean ± SEM. Statistical analysis were performed by a 2-way ANOVA followed by a Bonferroni post hoc test. ** *p* < 0.01; *** *p* < 0.001.

Since Cdk5 activation in LPS-stimulated macrophages is shown to suppress Il-10 production [16] our aim was to investigate whether Cdk5 inhibition with roscovitine reduces Il-10 expression in inflammatory macrophages. We pre-treated bone marrow-derived macrophages (BMDMs) from wild-type mice with either vehicle (DMSO) or 0.16 µM roscovitine and then exposed them to LPS for 4 h. In line with previous findings [20], we could show that LPS treatment alone increased the mRNA expression of the anti-inflammatory cytokine *Il-10* to prevent exaggerated inflammatory responses (Figure 1A). Interestingly, the co-treatment of LPS and roscovitine synergistically enhanced the *Il-10* expression when compared to macrophages stimulated with LPS alone (Figure 1A). The roscovitine treatment did not change the *Cdk5* mRNA expression (Supplementary Figure S1A). To further confirm these findings, we generated macrophage-specific *Cdk5* knockout mice (hereafter referred as *Cdk5*^LysMCre^) using the Cre/loxP system as previously described [20]. We isolated bone marrow cells from *Cdk5*^LysMCre^ mice and from their littermate wild-type controls (hereafter referred as *Cdk5*^flox^) and differentiated them into BMDMs. In our previous study, we confirmed the Cdk5 deletion at mRNA and protein level in bone marrow-derived macrophages cultured in vitro [20]. Consistent with our findings from the Cdk5 inhibition experiments, the LPS treatment of macrophages isolated from *Cdk5*^flox^ and *Cdk5*^LysMCre^ mice increased the mRNA and protein expression of Il-10, respectively (Figure 1B,C). Furthermore, deletion of macrophage-specific *Cdk5* resulted in significantly higher Il-10 mRNA and protein expression compared with *Cdk5*^flox^ macrophages after LPS treatment (Figure 1B,C). Moreover, Il-10 is shown to mediate the suppression of the pro-inflammatory cytokine Il-12 [21]. *Il-12* mRNA expression was significantly reduced, and Il-12 protein showed a strong trend of reduction in *Cdk5*^LysMCre^ macrophages compared with macrophages isolated from *Cdk5*^flox^ mice after LPS stimulation (Figure 1D, Supplementary Figure S1C).

**Figure 2:**
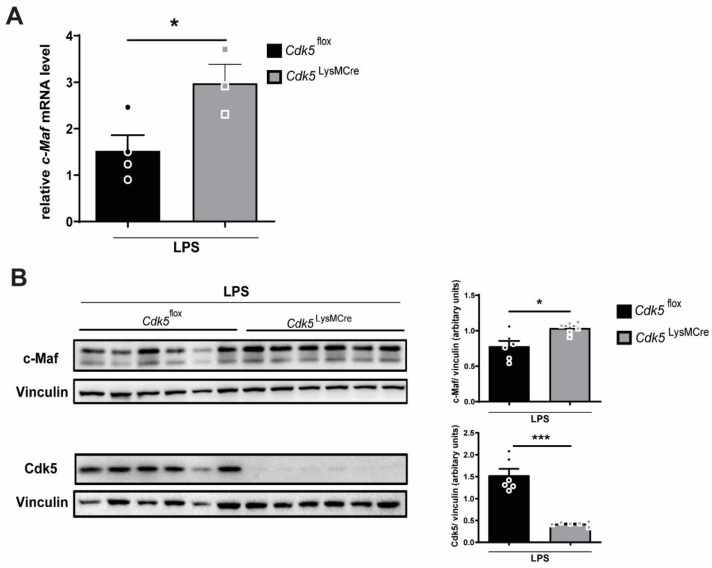
Genetic *Cdk5* deletion in bone marrow-derived macrophages increases c-Maf after LPS stimulation. BMDMs from *Cdk5*^flox^ and *Cdk5*^LysMCre^ mice were stimulated with PBS (Co) or LPS (100 ng/mL) for 4 h and (**A**) relative *c-Maf* mRNA expression was measured by qRT-PCR and (**B**) c-Maf protein (42 kDa), Cdk5 (30 kDa) and vinculin (117 kDa) as loading control were detected by immunoblot (left) and quantified (right). Results are depicted as mean ± SEM. Statistical analysis were performed by a normality test, followed by a two-tailed unpaired Student’s *t* test. * *p* < 0.05; *** *p* < 0.001.

Recently, it was reported that c-Maf promotes Il-10 with an inhibitory effect on Il-12 production [9]. Moreover, macrophages from *c-Maf* knockout mice showed reduced expression of Il-10 at mRNA and protein level [10,11]. In contrast, the Il-12 expression was significantly enhanced in c-Maf knockout macrophages [10]. Because Il-10 expression was induced both after inhibition and deletion of Cdk5 in macrophages upon inflammatory stimuli (Figure 1A–C), we examined c-Maf, an upstream regulator of Il-10 and Il-12. [9,10]. Our results exhibited a significant increase in both mRNA and protein levels of c-Maf in macrophages isolated from *Cdk5*^LysMCre^ mice compared with macrophages from *Cdk5*^flox^ mice upon LPS stimulation (Figure 2A,B). This is in line with the inhibition of Cdk5 by roscovitine leading to increased *c-Maf* expression (Supplementary Figure S1B).

Taken together, our findings suggest that upon inflammatory stimuli macrophage-specific deletion of *Cdk5* enhances the expression of anti-inflammatory cytokine Il-10, whereas the expression of the pro-inflammatory cytokine Il-12 is reduced. We further showed that Cdk5 mediates these effects through expression of c-Maf.

### 2.2. Genetic Deletion of Cdk5 in Macrophages Enhances Il-10 Levels through C-Maf in Mouse Lungs during Endotoxemia

**Figure 3:**
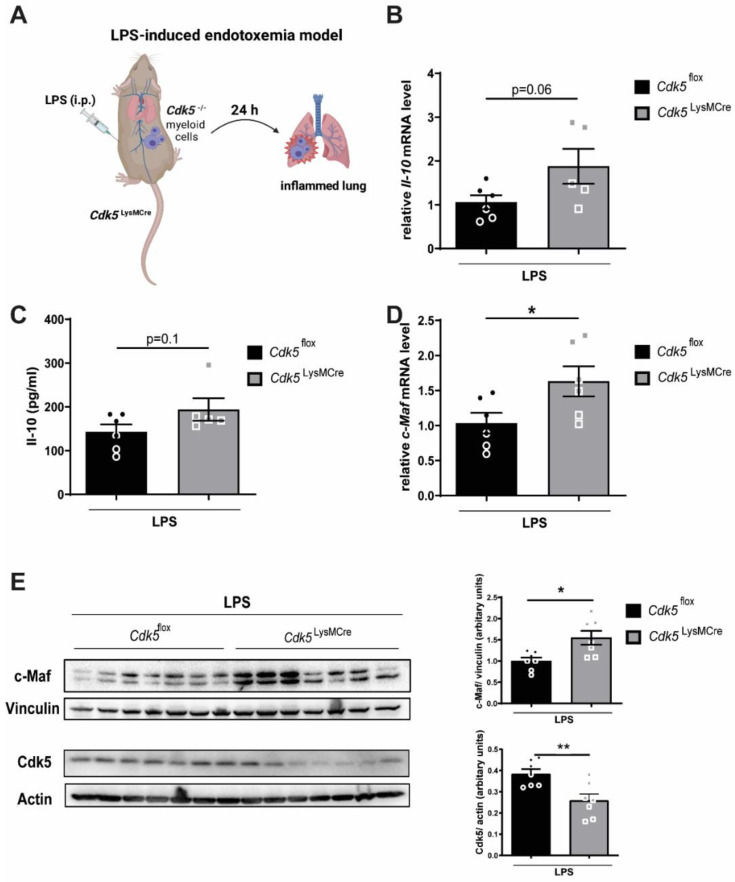
Myeloid-specific *Cdk5* deletion leads to increased c-Maf and hence Il-10 levels in the lung of mice during LPS-induced endotoxemia. (**A**) Schematic representation of the experimental setup for LPS-induced endotoxemia (LPS dose: 15 mg/kg) created with *BioRender.com.* (**B**) *Il-10* mRNA expression in homogenized lung tissue from endotoxemic *Cdk5*^flox^ and *Cdk5*^LysMCre^ mice after 24 h was determined by qRT-PCR (**C**) Il-10 concentration was measured in bronchoalveolar lavage (BAL) of endotoxemic *Cdk5*^flox^ and *Cdk5*^LysMCre^ mice after 24 h by ELISA (**D**,**E**) homogenized lung tissue from endotoxemic *Cdk5*^flox^ and *Cdk5*^LysMCre^ mice after 24 h was analyzed for (**D**) *c-Maf* mRNA expression and (**E**) c-Maf protein expression. c-Maf protein (42 kDa) and vinculin (117 kDa) as loading control as well as Cdk5 (30 kDa) and Actin (42 kDa) as loading control were detected by immunoblot (left) and quantified (right). Results are depicted as mean ± SEM. Statistical analysis were performed by a normality test, followed by a two-tailed unpaired Student’s *t* test. * *p* < 0.05; ** *p* < 0.01.

In LPS-mediated endotoxemia, Il-10 is well-known to exert an immunoregulatory function, and mice lacking Il-10 production showed increased lethality in response to LPS [4]. Progressive endotoxemia can result in an acute respiratory distress syndrome (ARDS) and acute lung inflammation (ALI) [6,7,22] and treatment with Il-10 is shown to improve survival in ALI mouse models [8]. Our in vitro data revealed that the deletion of *Cdk5* in macrophages enhances the anti-inflammatory cytokine Il-10. Therefore, our aim was to investigate whether *Cdk5* deletion in myeloid cells modulates Il-10 production during endotoxemia. Thus, we induced LPS-mediated endotoxemia in *Cdk5*^flox^ and *Cdk5*^LysMCre^ mice and investigated the lung and the bronchoalveolar lavage (BAL) of *Cdk5*^flox^ and *Cdk5*^LysMCre^ mice after 24 h (Figure 3A). We observed a trend towards increased *Il-10* mRNA in the lung tissue (Figure 3B) and Il-10 protein in the BAL (Figure 3C) of *Cdk5*^LysMCre^ mice when compared to *Cdk5*^flox^ littermate controls. The total number of macrophages in the BAL was not changed (Supplementary Figure S2A). Furthermore, the c-Maf mRNA and protein expression was significantly increased in the lung tissue from *Cdk5*^LysMCre^ when compared to the *Cdk5*^flox^ littermate controls upon LPS-induced endotoxemia (Figure 3D,E).

Together, these results suggest that the deletion of *Cdk5* in macrophages increases c-Maf expression in the lung tissue and thus induces the expression of the anti-inflammatory cytokine Il-10 during LPS-induced endotoxemia.

### 2.3. Cdk5^LysMCre^ Mice Show an Ameliorated Disease Severity during Endotoxemia

**Figure 4:**
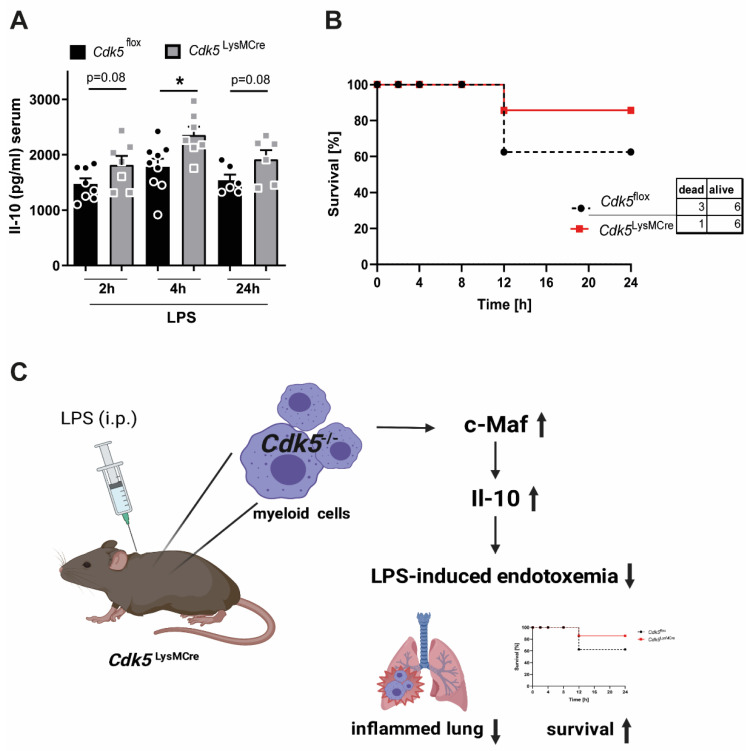
Myeloid-specific *Cdk5* deletion increases systemic Il-10 plasma levels, which leads to increased survival of *Cdk5*^LysMCre^ mice during LPS-induced endotoxemia. (**A**) Il-10 in plasma of *Cdk5*^flox^ and *Cdk5*^LysMCre^ mice was detected by ELISA during endotoxemia (LPS 15 mg/kg) after 2 h, 4 h and 24 h. (**B**) Survival of *Cdk5*^flox^ (n = 9) and *Cdk5*^LysMCre^ (n = 7) mice was monitored for 24 h during LPS-induced endotoxemia. (**C**) Schematic representation showing that the absence of Cdk5 in macrophages results in elevated c-Maf and Il-10 levels, thus contributing to an ameliorated anti-inflammatory response during endotoxemia. Created with *BioRender.com.* Results are depicted as mean ± SEM. Statistical analysis were performed by (**A**) a normality test, followed by a two-tailed unpaired Student’s *t* test, (**B**) Kaplan–Meier (Chi-square) *p* = n.s.; * *p* < 0.05.

The importance of Il-10 in maintaining survival during endotoxemia was shown by *Il-10* knockout animals and Il-10 injections [4,8]. Next, we wanted to examine whether myeloid-specific deletion of *Cdk5* in mice exposed to LPS-induced endotoxemia would enhance systemic plasma Il-10 levels and contribute to increased survival. To investigate this, we treated *Cdk5*^LysMCre^ and *Cdk5*^flox^ littermate control mice with LPS for 24 h and monitored their inflammatory status. Interestingly, the *Cdk5*^flox^ littermate control mice showed a significantly increased weight loss after 24 h endotoxemia (Supplementary Figure S2B). In addition, plasma Il-10 levels of *Cdk5*^LysMCre^ were significantly elevated 4 h after LPS injection and showed a trend toward increased plasma Il-10 levels at 2 h and 24 h of LPS-induced endotoxemia compared with their *Cdk5*^flox^ littermates (Figure 4A). Moreover, we analyzed pro-inflammatory cytokines and chemokines in the plasma of *Cdk5*^LysMCre^ and *Cdk5*^flox^ mice during LPS-induced endotoxemia, since in vitro studies using *c-Maf* knockout macrophages showed elevated levels of Ccl5 and Il-1a in addition to Il-10 [11]. This suggests that c-Maf contributes to a reduced overall inflammatory status. Consistent with these findings, we observed decreased levels of pro-inflammatory cytokines such as Il-1a, Ccl5 (Rantes), Mcp1, Ccl3/Ccl4 (Mip1a/b), as well as Tnf-α in *Cdk5*^LysMCre^ mice compared with *Cdk5*^flox^ littermates during LPS-induced endotoxemia (Supplementary Figure S3A–F).

The total number of circulating white blood cells, red blood cells, lymphocytes, monocytes, and granulocytes in the blood of *Cdk5*^LysMCre^ was not changed after 24 h endotoxemia compared to *Cdk5*^flox^ mice (Supplementary Figure S4A–E). Interestingly, *Cdk5*^LysMCre^ mice showed a trend toward improved survival when compared with *Cdk5*^flox^ littermates during LPS-induced endotoxemia (Figure 4B). These results support our hypothesis that deletion of Cdk5 in macrophages contributes to an induction in Il-10 and thus increased survival after LPS-induced endotoxemia.

In summary, our data reveal a previously unknown role of Cdk5 in myeloid cells in the regulation of inflammatory processes during LPS-induced endotoxemia. Genetic deletion of *Cdk5* in myeloid cells increases c-Maf and hence Il-10 levels during LPS-induced endotoxemia, contributing to reduced lung inflammation and increased survival (Figure 4C).

## 3. Discussion

In this study, we investigated whether the deletion of *Cdk5* in macrophages regulates Il-10 production through c-Maf during LPS-induced endotoxemia. In primary bone marrow-derived macrophages, we were able to show that the deficiency of *Cdk5* results in enhanced c-Maf and Il-10 levels. These findings were confirmed in vivo during LPS-induced endotoxemia, which can progress to an acute lung injury. There, we observed increased c-Maf and enhanced Il-10 production in the lungs of *Cdk5*^LysMCre^ mice compared to *Cdk5*^flox^ littermate controls. This was accompanied by increased systemic plasma Il-10 levels, while the pro-inflammatory cytokines and chemokines, Il-1a, Ccl5, Mcp1, Ccl3/Ccl4 (Mip1a/b), and Tnf-α were reduced in the plasma of *Cdk5*^LysMCre^ mice, resulting in a reduced disease severity.

The Cdk5 activator p35 (Cdk5r1) and c-Maf were both independently shown to regulate Il-10 production in macrophages [9,10,11,16]. However, we demonstrated for the first time that Cdk5 regulates Il-10 production in macrophages through c-Maf.

Roscovitine is a small molecule Cdk inhibitor that was shown to increase Il-10 expression at a concentration of 5 µM in primary macrophages [16]. At high concentrations, roscovitine also inhibits other kinases such as Cdk1 (IC50 = 0.65 µM), Cdk2 (IC50 = 0.7 µM), Cdk7 (IC50 = 0.46 µM) and Cdk9 (IC50 = 0.6 µM), therefore we used a concentration of 0.16 µM that specifically inhibits Cdk5 (IC50 = 0.16 µM) (selleckchem webpage) and observed enhanced *Il-10* expression after LPS-mediated TLR4 stimulation. In addition, Seok et al. showed enhanced *Il-10* expression after LPS stimulation in a transient *Cdk5* siRNA knockdown [16]. Consistent with this, our conditional *Cdk5* macrophage knockout confirmed our findings with roscovitine and similarly increased Il-10 and c-Maf expression after LPS stimulation when compared to the respective control. Seok et al. focused on p35 with total *p35* KO animals as an activator of Cdk5 and investigated the Il-10 production after LPS stimulation. We took the previously mentioned authors’ published global gene expression analysis on the Gene Expression Omnibus database of LPS-stimulated macrophages from wild-type and *p35* KO mice (GEO: GSE63443) and found that *c-Maf* is induced in *p35* KO macrophages compared to wild-type macrophages [16] further supporting our finding that Cdk5 itself regulates c-Maf.

It has been reported that c-Maf is stimulated by LPS, increases anti-inflammatory Il-10 production in macrophages, and reduces the pro-inflammatory cytokine Il-12 [9,10]. This is in line with our results showing that LPS-stimulated primary macrophages have enhanced c-Maf expression, hence induced Il-10 expression and reduced Il-12 expression. Interestingly, *Cdk5* deletion in macrophages increased significantly both c-Maf and Il-10 compared with macrophages isolated from *Cdk5*^flox^ mice after LPS stimulation. This suggests that during inflammation Cdk5 inhibits the LPS-mediated c-Maf-Il-10 axis. Therefore, inhibition of Cdk5 could potentiate the anti-inflammatory effect of Il-10 and ameliorate the pro-inflammatory effect of Il-12, suggesting better resolution of inflammation in inflammatory diseases. In agreement with our findings, roscovitine enhances the resolution of inflammation in a mouse model of arthritis and reduced lung inflammation [23,24]. In our previous study, we also showed that roscovitine treatment or macrophage-specific *Cdk5* deletion enhanced the potent anti-inflammatory effects of glucocorticoids during inflammation [20]. Our findings, and the previously published data, support the idea that inhibition of Cdk5 may prove useful in enhancing anti-inflammatory effects in inflammatory diseases.

*Cdk5*^LysMCre^ macrophages have increased c-Maf mRNA and protein expression during inflammatory stimuli in comparison to *Cdk5*^flox^ macrophages. However, the underlying mechanism by which Cdk5 regulates c-Maf expression remains to be elucidated. A potential mechanism and common mediator could be glycogen synthase kinase-3 (Gsk3). Gsk3 interacts with c-Maf and regulates the function of c-Maf by phosphorylation [25]. Furthermore, it is shown that the inhibition of Cdk5 in neurons activates Gsk3 [26,27]. Therefore, a possible mediator between Cdk5 and c-Maf could be Gsk3, which might be induced upon Cdk5 deletion and regulates c-Maf function. However, this needs to be investigated in further experiments.

In the LPS-induced endotoxemia mouse model we observed significant enhanced c-Maf mRNA as well as protein expression in the lung of *Cdk5*^LysMCre^ mice compared to the *Cdk5*^flox^ littermate controls. We further detected a mild increase of Il-10 production in the plasma and lung tissue as well as in the BAL of *Cdk5*^LysMCre^ mice during endotoxemia. One possible explanation for the fact that we observed only a slight increase in BAL Il-10 could be that *Cdk5* is deleted only in the myeloid lineage through the Cre/loxP system and for this reason the Cdk5 expression can be detected still with a reduced intensity. Other immune cells, such as T-cells and B-cells, which are also known to produce Il-10 in response to inflammatory stimuli, may further contribute to the Il-10 production during the inflammatory response [28]. Seok et al. reported a nearly 4-fold increase in plasma Il-10 after endotoxemia in full *p35* KO mice [16]. In comparison, our data shows only a mild increase in Il-10 levels, which can be explained by the myeloid-specific *Cdk5* deletion compared with the total deletion of the Cdk5 activator *p35* from Seok et al. In addition, the enhanced c-Maf level resulted in significantly reduced Ccl5, Mcp1, Ccl3 (Mip1a), and Tnf-α plasma levels and tended to reduce Il-1a and Ccl4 (Mip1b) plasma levels in the *Cdk5*^LysMCre^ mice after endotoxemia, suggesting better resolution of inflammation. This is consistent with previously reported in vitro data in primary c-Maf knockout macrophages, where enhanced *Ccl5* and *Il-1* levels were observed [11].

In summary, we report a novel mechanism of Cdk5 regulating c-Maf expression and hence Il-10 expression in macrophages under inflammatory conditions. In detail, both inhibition and deletion of myeloid-specific *Cdk5* under inflammatory conditions increases c-Maf and Il-10 levels, decreases pro-inflammatory cytokine levels, and thus contributes to the resolution of inflammation and improved survival (Figure 4C). Our data suggests that inflammatory diseases could be treated with the specific Cdk5-inhibitory dose of roscovitine to promote anti-inflammatory effects. Roscovitine is already used to enhance the resolution of inflammation in the lung and in arthritis [23,24,29]. Moreover, a clinical trial (phase II NTC 02649751) was investigating the function of roscovitine in the chronic inflammatory lung disease cystic fibrosis by monitoring pro- (TNF-α) and anti-inflammatory (IL-10) parameters, among others. The data from this clinical trial are so far not published. Our data therefore supports the approach of treating inflammatory diseases, in particular sepsis and acute lung injury, with roscovitine or other drugs that inhibit Cdk5.

## 4. Materials and Methods

### 4.1. Mice

*Cdk5*^tm1Bibb^ (C57BL/6) mice (hereafter named as *Cdk5*^flox^) were kindly provided by Prof. Dr Johanna Pachmayr (Paracelsus Medical Private University, Austria [30]). *Cdk5*^flox^ mice were crossed with transgenic Lyz2^tm1(cre)lfo/J^ (C57BL/6) mice (hereafter named as LysMCre) to generate *Cdk5*^LysMCre^ mice. Male and female *Cdk5*^LysMCre^ mice and littermate controls (*Cdk5*^flox^) at the age of 8–13 weeks were used for experiments. Animals were housed at the Centre of Biomedical Research (ZBMF) at Ulm University. Animals were maintained under standardized conditions and were equally distributed in terms of age and body weight. This study was approved by the federal authorities for animal research of the Regierungspräsidium Tübingen, Baden-Wuerttemberg, Germany, and performed in adherence with the National Institutes of Health Guidelines on the Use of Laboratory Animals and the European Union “Directive 2010/63 EU on the protection of animals used for scientific purposes”. The survival analysis was performed by termination criteria in a premorbidity state assigned by the local authorities.

### 4.2. In Vivo Mouse Model

Endotoxemia was performed as previously described [31]. In short, LPS (Sigma-Merck, Germany; L2880, 15 mg/kg) was applied intra peritoneal to male and female *Cdk5*^flox^ and *Cdk5*^LysMCre^ mice at the age of 10–16 weeks for 24 h. Immediately after sacrificing the mice, bronchoalveolar lavage (BAL) was collected by tracheal cannulation using 1 mL of cold PBS + 10 mM EDTA. BAL was centrifuged at 300× *g* for 15 min at 4 °C and the pellet was used for flow cytometry analysis and supernatant was used to perform an Il-10 ELISA. Blood was taken retrobulbar or through puncture of the tail vein.

### 4.3. Measurements of Cytokine and Chemokine Concentrations

Bio-Plex Pro Mouse Cytokine 23-plex Assay (Group I) (Biorad, Germany) was used to measure 23 cytokines, chemokines, and growth factors simultaneously in the plasma. The Bio-Plex Assay was conducted according to the manufacturer´s protocol. The assay was performed with Bio-Plex 200 machine (Biorad, Germany) and analyzed with the Bio-Plex Manager TM 6.1 software (Biorad, Germany).

### 4.4. Blood Cell Anaylsis

Blood cells were analyzed shortly after the collection of blood with animal blood counter from scil vet. Fifteen microliters of blood were used for the measurement.

### 4.5. Flow Cytometry

Cells from the BAL were blocked with Fc block (CD16/32) (1:200) (ebioscience) for 20 min, stained for CD45-APC (1:200) (ebioscience) and F4/80-AF700 (1:200) (ebioscience) for 30 min and analyzed with BD LSRII (BD Biosciences) and FlowJo software (BD Biosciences).

### 4.6. Cell Culture

Primary bone marrow-derived macrophages (BMDMs) were isolated from humerus, femur and tibia of 8–13 weeks old littermate wild-type *Cdk5*^flox^ and *Cdk5*^LysMCre^ mice. Bone marrow was flushed out and cells were cultured until day 7 in DMEM (D5671, Sigma-Merck, Germany) supplemented with 10% fetal bovine serum (FBS, F7524, Sigma-Merck, Germany), 30% L929-cell conditioned medium, 1% penicillin/streptomycin (P0781, Sigma), 1% L-glutamin (G7513, Sigma-Merck, Germany), 1% sodium pyruvate (S8636, Sigma) at 37 °C and 5% CO_2_. For roscovitine experiments BMDMs from wild-type mice (C57BL/6) were pre-treated for 30 min with DMSO (as vehicle) or 0.16 µM roscovitine (Seliciclib, CYC202, Selleckchem, Houston, TX, USA). All BMDMs were treated with PBS as control or LPS (100 ng/mL, L6529, Sigma) for the indicated durations.

### 4.7. Rna Isolation and Quantitative Rt-Pcr

Primary macrophages were washed with 1× PBS and then scraped in RLT (Qiagen, Hilden, Germany) + 10 µL β-mercaptoethanol / ml buffer. RNA was isolated using the RNeasy^®^ Mini Kit (Qiagen, Hilden, Germany) according to the manufacturer´s protocol. The 1000 ng RNA was reversed transcribed to cDNA by using Superscript II^®^ (Superscript^®^ Reverse Transcriptase, Invitrogen, Waltham, MA, USA). Quantitative RT-PCR (qRT-PCR) was performed with the ViiA™ 7 Realtime PCR System (Life technologies, USA) using Platinum SYBR Green (Invitrogen, Waltham, MA, USA). For analysis the QuantStudio Realtime-PCR software and the ΔΔC_T_ method was used. *β*-Actin and *Ribosomal protein* L. (*Rpl*) served as housekeeping genes. The specific primers (5′ to 3′ direction) were obtained from Sigma with the sequences listed as following: Actin forward GCACCAGGGTGTGATGGTG, reverse CCAGATCTTCTCCATGTCGTCC, Rpl forward CCTGCTGCTCTCAAGGTT, reverse TGGCTGTCACTGCCTGGTACTT, II-10 forward CAGAGCCACTGCTCCTAGA, reverse TGTCCAGCTGGTCCTTTGTT, c-Maf forward CAACGGCTTCCGAGAAAAC, reverse GTAGAGGAGTCCCTTCCCTAC, Il-12 forward TCAGAATCACAACCATCAGCA, and reverse CTCGCCATTATGATTCAGAGACT.

### 4.8. Interleukin-10 ELISA

Supernatant from the BAL was undiluted analyzed. For cells: 1 × 10^6^ BMDMs were seeded in 6 cm dishes (cell culture coated) and after indicated time points the supernatant was harvested and stored at −80 °C until the Il-10 ELISA (DY417, R&D system, Minneapolis, MN, USA) was performed according to the manufacturer´s protocol.

### 4.9. Immunoblot Analysis

BMDMs were washed with 1× ice-cold PBS and lysed directly on the dishes with ice-cold 1× RIPA buffer. 1× PhosphoStop (Roche, Switzerland) and 1× protease inhibitor cocktail (Roche, Switzerland) were added to both buffers. The lysates were centrifuged at 14,000 rpm, 4 °C for 10 min. The protein concentration was determined using the Pierce^®^ BCA Protein Assay Kit (Thermo Scientific, Waltham, MA, USA) according to the manufacturer’s instructions. For immunoblot analysis, protein samples were adjusted to 20–25 µg protein with lysis or RIPA buffer and boiled in 1× Laemmli buffer (with 10 µL/ mL β-mercaptoethanol) at 95 °C for 5 min. Equal protein amounts were separated on 10% SDS- PAGE gels and subsequently electrotransferred onto nitrocellulose membranes (Biorad, Germany) using the Tank Blot System (Biorad). The membranes were blocked with 5% BSA (Sigma-Merck, Germany) in tris-buffered saline with tween20 (TBS-T) for 1h at RT and probed over night at 4 °C with primary antibodies against c-Maf (Proteintech #55013–1-AP, 1:500), Cdk5 (cell signaling #2506, 1:1000), Il-12 (R&D #AF-419-SP, 1:500), vinculin (Santa Cruz Biotechnology #sc-73614, 1:1000) and β-Actin (Sigma-Merck, Germany, 1:1000). After washing with TBS-T for 30 min, membranes were incubated with horseradish peroxidase-coupled goat anti-mouse (Dako, Santa Clara, CA, USA) or goat anti-rabbit (Life Technologies, Carlsbad, CA, USA) antibodies for 1h at RT. For visualization the LuminataTM Forte Western HRP Substrate (Milipore, Sigma-Merck, Germany) and the ChemiDocTM MP Imaging System (Biorad, Germany) was used. Quantification was performed with ImageJ software. Vinculin and β-Actin served as loading controls.

### 4.10. Statistical Analysis

The statistical analysis was carried out with GraphPad Prism 7 software. All data are shown as mean ± SEM. Outlying sample exclusion criteria were performed with GraphPad Prism Outlier Calculator. All data were tested using a normality test, followed by a two-tailed unpaired Student’s t test or 2-way ANOVA followed by a Bonferroni post hoc test or Kaplan–Meier (Chi-square). In comparison, mean values which show significance are indicated as follows: * *p* ˂ 0.05; ** *p* ˂ 0.01; *** *p* ˂ 0.001; ns: not significant.

## Data Availability

Not applicable.

## Supplementary Materials

The following are available online at https://www.mdpi.com/article/10.3390/ijms22179648/s1.

## References

[1] Moore K.W., Malefyt R.d.W., Coffman R.L., O’Garra A. (2001). Interleukin-10 and the interleukin-10 receptor. Annu. Rev. Immunol..

[2] Mosser D.M., Edwards J.P. (2008). Exploring the full spectrum of macrophage activation. Nat. Rev. Immunol..

[3] Kühn R., Löhler J., Rennick D., Rajewsky K., Müller W. (1993). Interleukin-10-deficient mice develop chronic enterocolitis. Cell.

[4] Berg D.J., Kuhn R., Rajewsky K., Muller W., Menon S., Davidson N., Grunig G., Rennick D. (1995). Interleukin-10 is a Central Regulator of the Response to LPS in Murine Models of Endotoxic Shock and the Shwartzman Reaction but not Endotoxin Tolerance. J. Clin. Investig..

[5] Orraine L., Are B.W., Atthay A.M. (2000). The acute respiratory distress syndrome. N. Engl. J. Med..

[6] Bernard G.R., Artigas A., Brigham K.L., Carlet J., Falke K., Hudson L., Lamy M., Legall J.R., Morris A., Spragg R. (1994). Intensive Care Medicine Report of the American-European consensus conference on ARDS: Definitions, mechanisms, relevant outcomes and clinical trial coordination. Am. J. Respir. Crit. Care Med..

[7] Herridge M.S., Cheung A.M., Tansey C.M., Matte-Martyn A., Diaz-Granados N., Al-Saidi F., Cooper A.B., Guest C.B., David Mazer C., Mehta S. (2003). One-Year Outcomes in Survivors of the Acute Respiratory Distress Syndrome. N. Engl. J. Med..

[8] Inoue G. (2000). Effect of interleukin-10 (IL-10) on experimental LPS-induced acute lung injury. J. Infect. Chemother..

[9] Cao S., Liu J., Chesi M., Bergsagel P.L., Ho I.-C., Donnelly R.P., Ma X. (2002). Differential Regulation of IL-12 and IL-10 Gene Expression in Macrophages by the Basic Leucine Zipper Transcription Factor c-Maf Fibrosarcoma. J. Immunol..

[10] Cao S., Liu J., Song L., Ma X. (2005). The Protooncogene c-Maf Is an Essential Transcription Factor for IL-10 Gene Expression in Macrophages. J. Immunol..

[11] Liu M., Tong Z., Ding C., Luo F., Wu S., Wu C., Albeituni S., He L., Hu X., Tieri D. (2020). Transcription factor c-Maf is a checkpoint that programs macrophages in lung cancer. J. Clin. Investig..

[12] Nishizawa M., Kataoka K., Gotot N., Fujiwara K.T., Kawai S. (1989). v-maf, a viral oncogene that encodes a “leucine zipper” motif (avian retrovirus/transformation/DNA binding protein). Proc. Natl. Acad. Sci. USA.

[13] Ding Wu Y., Levy D.E., Ochando J.C., Jiangnan Xu J.S., Yang Y., Qiu G., Lal G. (2009). c-Maf Regulates IL-10 Expression during Th17 Polarization. J. Immunol. Ref..

[14] Apetoh L., Quintana F.J., Pot C., Joller N., Xiao S., Kumar D., Burns E.J., Sherr D.H., Weiner H.L., Kuchroo V.K. (2010). The aryl hydrocarbon receptor interacts with c-Maf to promote the differentiation of type 1 regulatory T cells induced by IL-27. Nat. Immunol..

[15] Ma X., Yan W., Zheng H., Du Q., Zhang L., Ban Y., Li N., Wei F. (2015). Regulation of IL-10 and IL-12 production and function in macrophages and dendritic cells. F1000Research.

[16] Na Y.R., Jung D., Gu G.J., Jang A.R., Suh Y.H., Seok S.H. (2015). The early synthesis of p35 and activation of CDK5 in LPS-stimulated macrophages suppresses interleukin-10 production. Sci. Signal..

[17] Pareek T.K., Keller J., Kesavapany S., Pant H.C., Iadarola M.J., Brady R.O., Kulkarni A.B. (2005). Cyclin-dependent kinase 5 activity regulates pain signaling. Proc. Natl. Acad. Sci. USA.

[18] Rosales J.L., Ernst J.D., Hallows J., Lee K.Y. (2004). GTP-dependent secretion from neutrophils is regulated by Cdk5. J. Biol. Chem..

[19] Pareek T.K., Lam E., Zheng X., Askew D., Kulkarni A.B., Chance M.R., Huang A.Y., Cooke K.R., Letterio J.J. (2010). Cyclin-dependent kinase 5 activity is required for T cell activation and induction of experimental autoimmune encephalomyelitis. J. Exp. Med..

[20] Pfänder P., Fidan M., Burret U., Lipinski L., Vettorazzi S. (2019). Cdk5 deletion enhances the anti-inflammatory potential of GC-mediated GR activation during inflammation. Front. Immunol..

[21] Aste-Amezaga M., Ma X., Sartori A., Trinchieri G. (1998). Molecular Mechanisms of the Induction of IL-12 and Its Inhibition by IL-10. J. Immunol..

[22] Matthay M.A., Song Y., Bai C., Jones K.D., Edu M.M. (2013). The acute respiratory distress syndrome in 2013. Transl. Respir. Med..

[23] Rossi A.G., Sawatzky D.A., Walker A., Ward C., Sheldrake T.A., Riley N.A., Caldicott A., Martinez-Losa M., Walker T.R., Duffin R. (2006). Cyclin-dependent kinase inhibitors enhance the resolution of inflammation by promoting inflammatory cell apoptosis. Nat. Med..

[24] Hoogendijk A.J., Roelofs J.J.T.H., Duitman J., van Lieshout M.H.P., Blok D.C., van der Poll T., Wieland C.W. (2012). R-roscovitine reduces lung inflammation induced by lipoteichoic acid and Streptococcus pneumoniae. Mol. Med..

[25] Rocques N., Abou Zeid N., Sii-Felice K., Lecoin L., Felder-Schmittbuhl M.P., Eychène A., Pouponnot C. (2007). GSK-3-Mediated Phosphorylation Enhances Maf-Transforming Activity. Mol. Cell.

[26] Paglini G., Pigino G., Kunda P., Morfini G., Maccioni R., Quiroga S., Ferreira A., Cáceres A. (1998). Evidence for the participation of the neuron-specific CDK5 activator P35 during laminin-enhanced axonal growth. J. Neurosci..

[27] Morfini G., Szebenyi G., Brown H., Pant H.C., Pigino G., DeBoer S., Beffert U., Brady S.T. (2004). A novel CDK5-dependent pathway for regulating GSK3 activity and kinesin-driven motility in neurons. EMBO J..

[28] Zhang H., Kuchroo V. (2019). Epigenetic and transcriptional mechanisms for the regulation of IL-10. Semin. Immunol..

[29] Duffin R., Leitch A.E., Sheldrake T.A., Hallett J.M., Meyer C., Fox S., Alessandri A.L., Martin M.C., Brady H.J., Teixeira M.M. (2009). The CDK inhibitor, R-roscovitine, promotes eosinophil apoptosis by down-regulation of Mcl-1. FEBS Lett..

[30] Liebl J., Zhang S., Moser M., Agalarov Y., Demir C.S., Hager B., Bibb J.A., Adams R.H., Kiefer F., Miura N. (2015). Cdk5 controls lymphatic vessel development and function by phosphorylation of Foxc2. Nat. Commun..

[31] Vettorazzi S., Bode C., Dejager L., Frappart L., Shelest E., Klaen C., Tasdogan A., Reichardt H.M., Libert C., Schneider M. (2015). ARTICLE Glucocorticoids limit acute lung inflammation in concert with inflammatory stimuli by induction of SphK1. Nat. Commun..

