# Supplementary material for: Deletion of Cdk5 in Macrophages Ameliorates Anti-Inflammatory Response during Endotoxemia through Induction of C-Maf and Il-10
Source: Int J Mol Sci. 2021 Sep 6;22(17):9648. doi: 10.3390/ijms22179648 (PMC8431799; doi:10.3390/ijms22179648)
Supplement: Supplementary file 1 [file ijms-22-09648-s001.zip › ijms-1302410-supplementary.pdf]

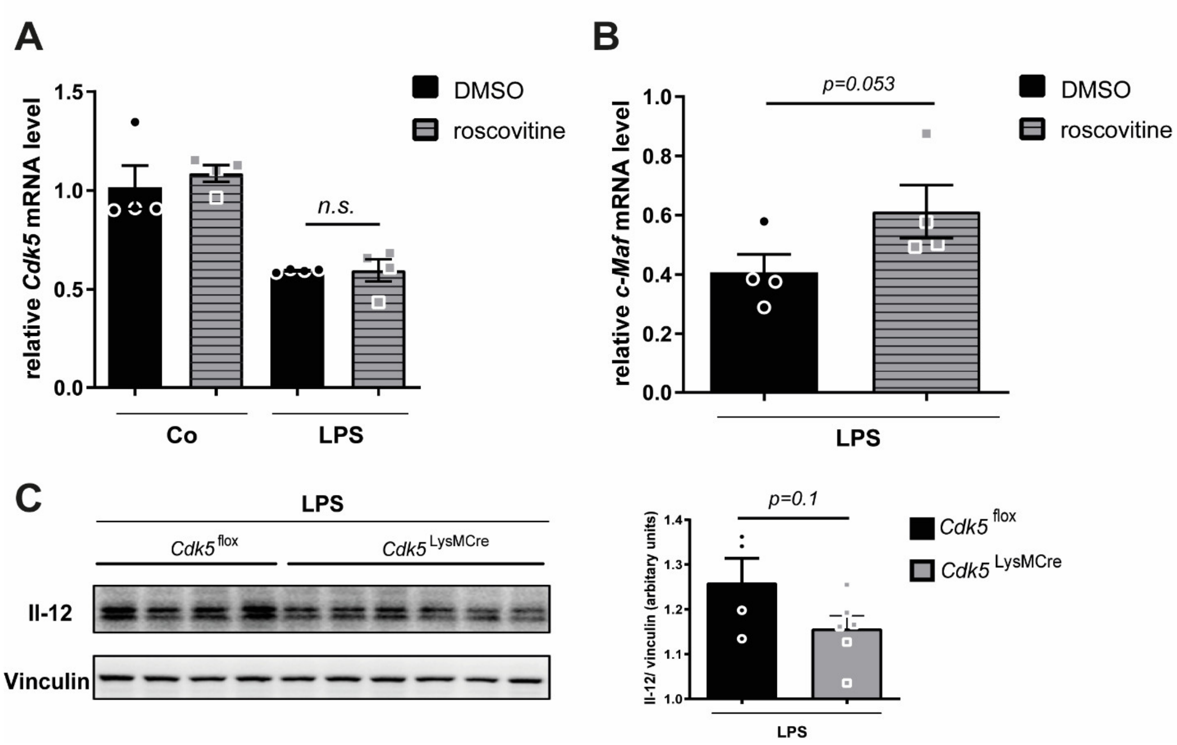

Supplementary Figure S1: (A) BMDMs derived from wild-type mice were 30 min pre-treated with 0.16 µM roscovitine, the specific concentration for Cdk5 inhibition or DMSO (vehicle control), and then stimulated 4 h with PBS (Co) or LPS (100 ng/ml) and relative expression of *Cdk5* mRNA was determined by qRT-PCR. (B) BMDMs derived from wild-type mice were 30 min pre-treated with 0.16 µM roscovitine or DMSO (vehicle control) and then stimulated 4 h with LPS (100 ng/ml) and relative expression of *c-Maf* mRNA was determined by qRT-PCR. (C) Il-12 (approx. 45kDa) protein and vinculin (117 kDa) as loading control were detected by immunoblot (left) and quantified (right). Results are depicted as mean ± SEM. Statistical analysis was performed by (A) a 2-way ANOVA followed by a Bonferroni post hoc test and (B) and (C) by a normality test, followed by a two-tailed unpaired Student's t test. n.s. not significant.

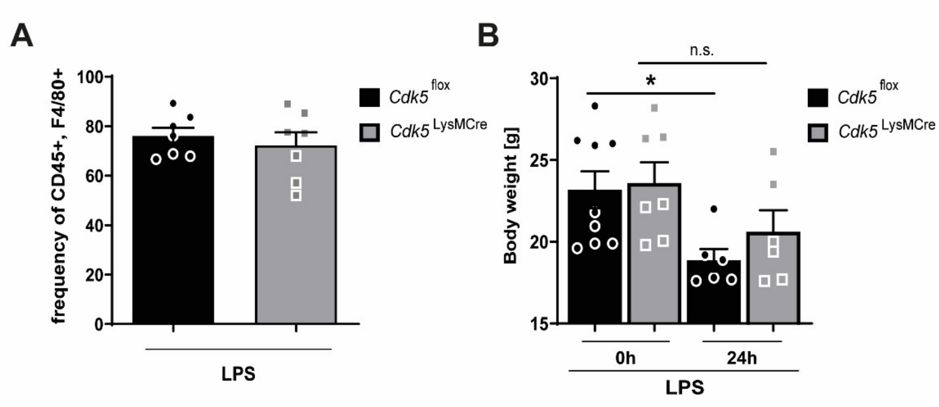

Supplementary Figure S2: (A) Flow cytometry analysis for macrophages ($CD45^+$, $F4/80^+$) from bronchoalveolar lavage (BAL) of *Cdk5*^{flox} and *Cdk5*^{LysMCre} mice after 24 h of LPS-induced endotoxemia. (B) Body weight of *Cdk5*^{flox} and *Cdk5*^{LysMCre} mice before and after 24 h of LPS-induced endotoxemia. Results are depicted as mean ± SEM. Statistical analysis was performed by a normality test, followed by a two-tailed unpaired Student's t test. * $p < 0.05$. n.s. not significant.

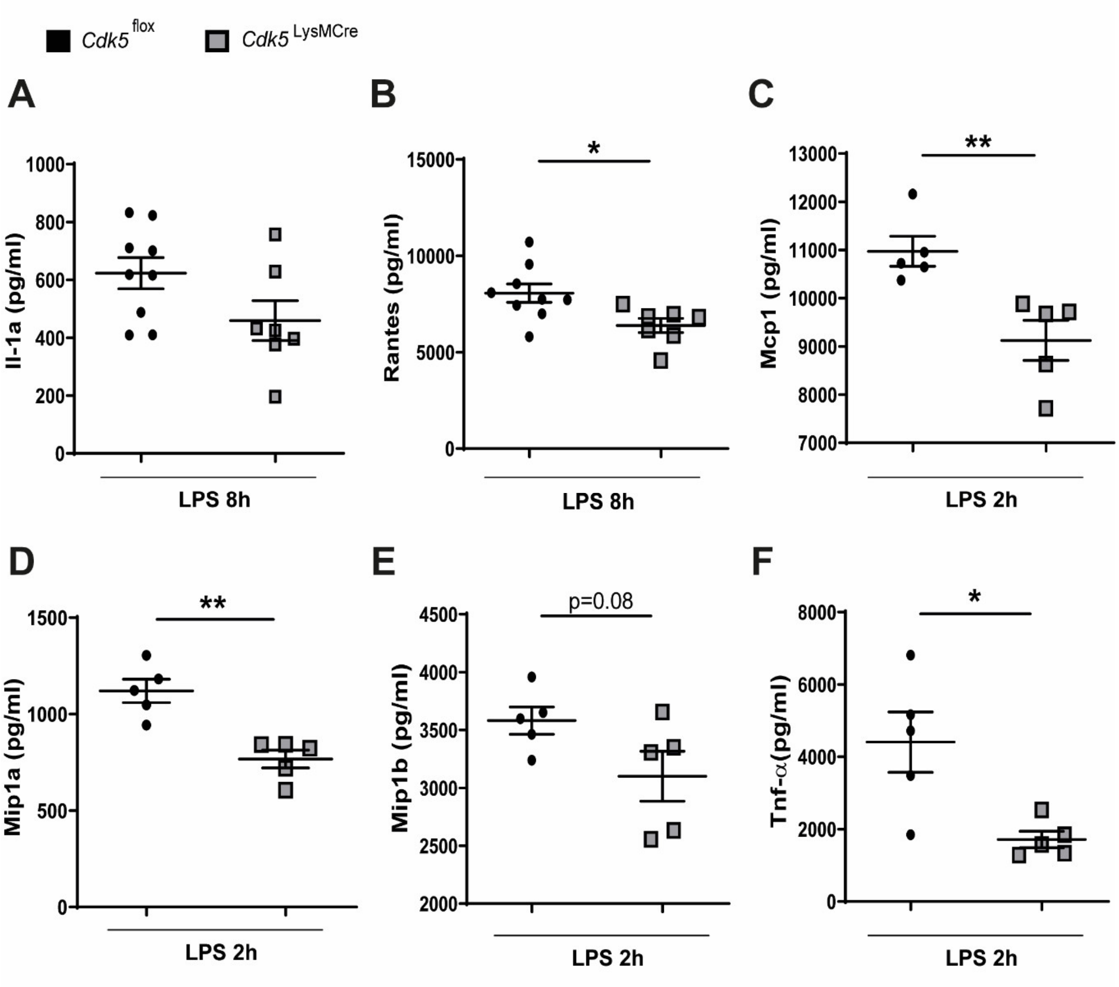

Supplementary Figure S3: Concentration of plasma (A) Il-1a (B) Ccl5 (Rantes), (C) Mcp1, (D) Mip1a, (E) Mip1b and (F) TNF-α protein from *Cdk5*^{flox} and *Cdk5*^{LysMCre} mice after LPS-induced endotoxemia at the indicated time points analysed with Bio-Plex Pro Mouse Cytokine 23-plex Assay. Results are depicted as mean ± SEM. Statistical analysis was performed by a normality test, followed bya two-tailed unpaired Student's t test. * $p < 0.05$; ** $p < 0.01$.

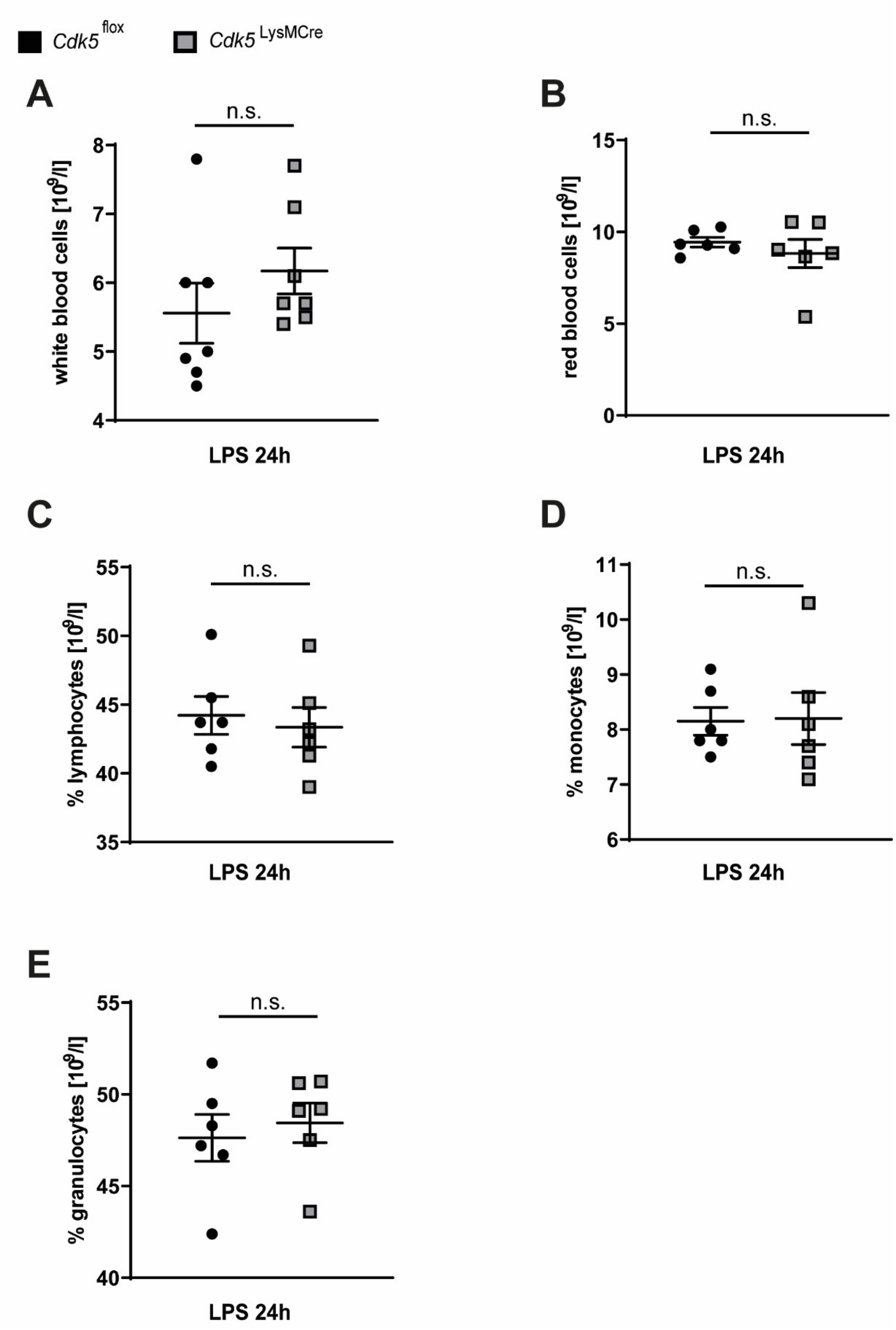

Supplementary Figure S4: Analysis of (A) white blood cells, (B) red blood cells, (C) lymphocytes, (D) monocytes and (E) granulocytes in the plasma from *Cdk5*^{flox} and *Cdk5*^{LysMCre} mice after 24 h LPS-induced endotoxemia were investigated with animal blood counter. Results are depicted as mean ± SEM. Statistical analysis was performed by a normality test, followed by a two-tailed unpaired Student's t test. n.s. not significant.
